# Enhanced killing of chordoma cells by antibody-dependent cell-mediated cytotoxicity employing the novel anti-PD-L1 antibody avelumab

**DOI:** 10.18632/oncotarget.9256

**Published:** 2016-05-09

**Authors:** Rika Fujii, Eitan R. Friedman, Jacob Richards, Kwong Y. Tsang, Christopher R. Heery, Jeffrey Schlom, James W. Hodge

**Affiliations:** ^1^ Laboratory of Tumor Immunology and Biology, Center for Cancer Research, National Cancer Institute, National Institutes of Health, Bethesda, MD, USA

**Keywords:** chordoma, programmed death-ligand 1 (PD-L1), antibody-dependent cell-mediated cytotoxicity (ADCC), cancer stem cells, immunotherapy

## Abstract

Chordoma, a rare bone tumor derived from the notochord, has been shown to be resistant to conventional therapies. Checkpoint inhibition has shown great promise in immune-mediated therapy of diverse cancers. The anti-PD-L1 mAb avelumab is unique among checkpoint inhibitors in that it is a fully human IgG1 capable of mediating antibody-dependent cell-mediated cytotoxicity (ADCC) of PD-L1-expressing tumor cells. Here, we investigated avelumab as a potential therapy for chordoma. We examined 4 chordoma cell lines, first for expression of PD-L1, and *in vitro* for ADCC killing using NK cells and avelumab. PD-L1 expression was markedly upregulated by IFN-γ in all 4 chordoma cell lines, which significantly increased sensitivity to ADCC. Brachyury is a transcription factor that is uniformly expressed in chordoma. Clinical trials are ongoing in which chordoma patients are treated with brachyury-specific vaccines. Co-incubating chordoma cells with brachyury-specific CD8^+^ T cells resulted in significant upregulation of PD-L1 on the tumor cells, mediated by the CD8^+^ T cells' IFN-γ production, and increased sensitivity of chordoma cells to avelumab-mediated ADCC. Residential cancer stem cell subpopulations of chordoma cells were also killed by avelumab-mediated ADCC to the same degree as non-cancer stem cell populations. These findings suggest that as a monotherapy for chordoma, avelumab may enable endogenous NK cells, while in combination with T-cell immunotherapy, such as a vaccine, avelumab may enhance NK-cell killing of chordoma cells *via* ADCC.

## INTRODUCTION

Chordoma is a rare bone cancer thought to arise from remnants of the embryonic notochord. Approximately 300 new cases per year are diagnosed in the United States, accounting for 20% of primary spine tumors and 1%-4% of all malignant bone tumors [1, 2]. Reported 5- and 10-year survival rates are about 70% and 40%, respectively, a reflection of the slow-growing nature of the disease. Surgery followed by radiotherapy is the standard of care for primary tumors. However, based on anatomic location and tumor size on presentation, a wide curative excision is rarely feasible [2]. Hence, incidence of disease recurrence is common and metastases have been reported in up to 40% of cases. Once metastases develop, median survival is about 1 year [1]. No treatment for advanced chordoma has been approved by the U.S. Food and Drug Administration (FDA), since chordoma is largely resistant to conventional chemotherapy [3]. Thus, there is an urgent need for novel therapeutic modalities for this disease.

Immunotherapy has become an important treatment option for chemotherapy-resistant cancers. Brachyury, a transcription factor uniformly expressed in chordoma [4], appears to be an oncogenic driver for this tumor type [5, 6]. In addition to being a diagnostic marker for chordoma, brachyury may be a potential target for treatment [7, 8]. Clinical trials of brachyury-specific vaccines are ongoing in chordoma patients. In addition, drugs that inhibit the immune checkpoints programmed cell death protein 1 (PD-1) and its major ligand, programmed death-ligand 1 (PD-L1), have shown clinical activity in diverse cancer types [9-11]. The FDA first approved a PD-1 inhibitor for melanoma; more recently, a PD-1 inhibitor was approved for lung cancer. Numerous clinical trials of several drugs targeting the PD-1/PD-L1 axis are ongoing in a range of cancers. Most of these PD-L1 antibodies are the IgG4 isotype and the Fc-modified IgG1 isotype, both of which inhibit the interaction of PD-1 on immune cells with PD-L1 on tumor cells [12]. Avelumab, a fully human IgG1 anti-PD-L1 monoclonal antibody (mAb), is the only anti-PD-L1 mAb that both induces antibody-dependent cell-mediated cytotoxicity (ADCC) and blocks the PD-1/PD-L1 pathway. Previously, our group reported that avelumab enhanced ADCC on several cancer cell lines expressing PD-L1 [13]. Other studies have shown that PD-L1 is expressed in chordoma cell lines and chordoma tissue samples [14, 15].

Here, for the first time, we demonstrate the potential of anti-PD-L1 antibody therapy for chordoma and report that (a) PD-L1 expression induced by IFN-γ increased the sensitivity of chordoma cells to lysis by natural killer (NK) cells *via* avelumab-mediated ADCC; (b) tumor antigen-specific CD8^+^ T cells indirectly induced PD-L1 expression on chordoma cells; (c) upregulated PD-L1 expression on chordoma cells indirectly induced by brachyury-specific CD8^+^ T cells increased the sensitivity of chordoma cells to avelumab-mediatedADCC; and (d) residential cancer stem cell (CSC) populations in chordoma cells were killed by avelumab-mediated ADCC to the same degree as non-CSC populations within the cells. Our findings suggest that while chordoma responds poorly to conventional therapies such as surgery, radiotherapy, and chemotherapy, immune-mediated therapy may have clinical benefit for some chordoma patients.

## RESULTS

### Treating chordoma cells with IFN-γ upregulates MHC-I and PD-L1 expression

**Figure 1:**
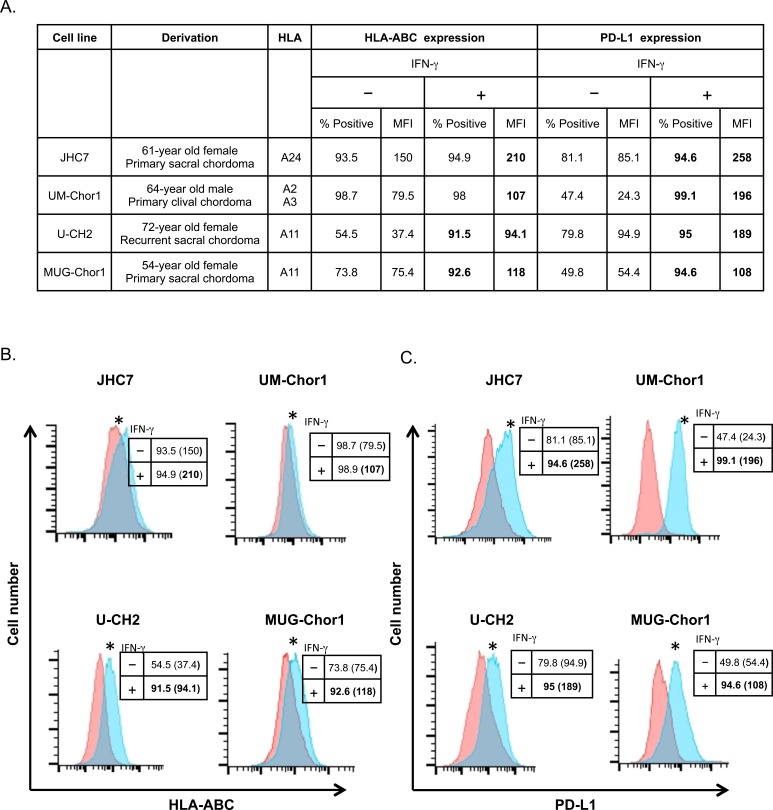
Treating chordoma cells with IFN-γ upregulates MHC-I and PD-L1 expression Chordoma cell lines established from 4 patients were treated with 50 ng/mL of IFN-γ or untreated as control for 24 h, then analyzed by flow cytometry. **A.** General characteristics of chordoma cell lines, surface expression of MHC-I (HLA-ABC) and PD-L1; percent positivity and MFI. **B.** Expression of HLA-ABC in chordoma cell lines treated with IFN-γ (blue histograms) or untreated (pink histograms). **C.** Expression of PD-L1 in chordoma cell lines treated with IFN-γ (blue histograms) or untreated (pink histograms). Insets: Numbers indicate % positive cells and MFI (parentheses). Values in bold denote an increase of > 10% relative to control cells. * = statistical significance over control (*P* < 0.05). This experiment was repeated at least 2 times with similar results.

It has been previously shown that IFN-γ upregulates MHC-I expression in cancer tissue [16, 17]. It has also been reported that IFN-γ upregulates PD-L1 expression in select chordoma cell lines [14, 15]. However, the potential of anti-PD-L1 antibody therapy in chordoma has not previously been shown. We first examined whether IFN-γ could modulate expression of MHC-I and PD-L1 in chordoma cell lines established from 4 chordoma patients [18-21]. All 4 cell lines expressed HLA-ABC and PD-L1, and both molecules were upregulated by IFN-γ in all 4 cell lines (Figure 1A). HLA-ABC expression in JHC7 cells treated with IFN-γ increased 1.4-fold relative to untreated controls (*P* < 0.001; Figure 1B). Similarly, IFN-γ treatment upregulated HLA-ABC expression (*P* < 0.001) in UM-Chor1 (1.35-fold), U-CH2 (2.52-fold), and MUG-Chor1 cells (1.56-fold). Moreover, IFN-γ significantly increased PD-L1 expression (*P* < 0.001) in JHC7 (3.03-fold), UM-Chor1 (8.06-fold), U-CH2 (1.99-fold), and MUG-Chor1 cells (1.99-fold; Figure 1C).

### Expression profiles of IFN-γ-induced genes in UM-Chor1 cells

To further examine the molecular consequences of treating chordoma cells with IFN-γ, we assessed IFN-γ-induced gene expression profiles of UM-Chor1 cells by microarray analysis (Supplemental Figure 1A). IFN-γ treatment upregulated genes in UM-Chor1 cells > 1.5-fold relative to untreated controls (*P* < 0.05). The highest upregulation was seen in gene *TP53INP2* (tumor protein p53 inducible nuclear protein 2), which regulates transcription and enhances starvation-induced autophagy [22]. The second highest upregulation was seen in gene *CEBPD* (CCAAT/enhancer binding protein [C/EBP] δ), which regulates proinflammatory gene expression [23, 24]. IFN-γ treatment downregulated some genes in UM-Chor1 cells > 1.5-fold relative to untreated controls (*P* < 0.05; (Supplemental Figure 1B). The most downregulated gene, *CLDN2*, has been identified as a tight junction-specific integral membrane protein [25] whose expression is affected by cytokines [26]. The second most downregulated gene, *PHACTR4I,* is a tumor suppressor gene that is mutated or downregulated in several cancers [27]. Supplemental Figure 1C shows the predicted pathway of IFN-γ-induced PD-L1 expression, as deduced from the results of microarray analysis. The transcription factor *CEBPD* is induced by IFN-γ, leading to inhibition of *MYC* and activation of *TLR9*, *IL10*, and *TNF*, and culminating in upregulation of PD-L1 (*CD274*) expression. Taken together, these results suggest that *CEBPD* is potentially involved in the pathway of IFN-γ-induced PD-L1 expression in chordoma cells.

### IFN-γ-treated chordoma cells showed increased sensitivity to NK-cell lysis *via* avelumab-mediated ADCC

**Figure 2:**
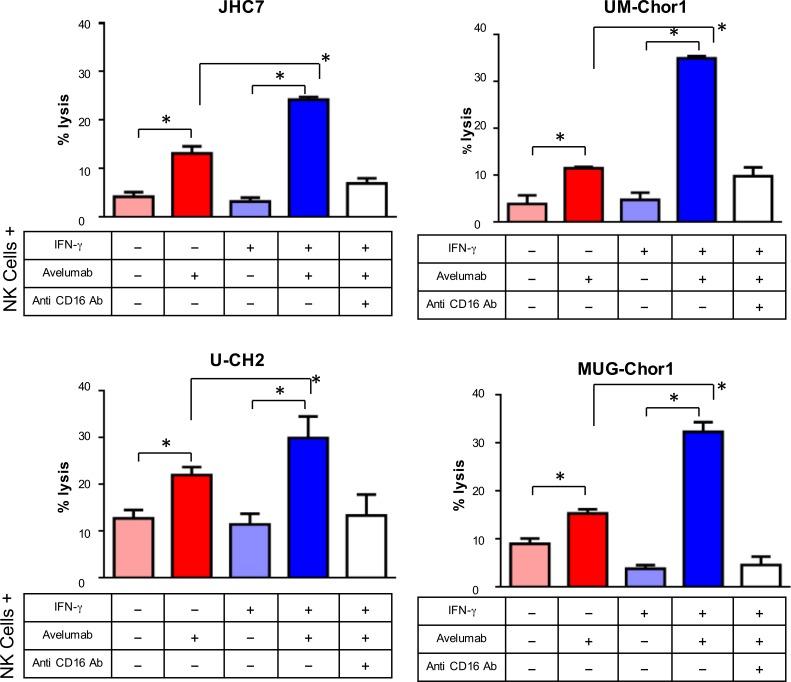
IFN-γ treatment increased chordoma cells' sensitivity to NK-cell lysis *via* avelumab-mediated ADCC ADCC assays were performed using 4 chordoma cell lines treated or untreated with IFN-γ, using normal donor NK cells as effector cells. Select groups of cells were incubated with avelumab and/or anti-CD16 antibody. Statistical analyses were done by Student's *t* test. * = *P* < 0.05, error bars indicate mean ± SD for triplicate measurements. This experiment was repeated at least 2 times with similar results.

Previously, our group reported that avelumab, an anti-PD-L1 antibody, enhanced NK cell-mediated lysis *via* ADCC on several cancer cell lines that express PD-L1 [13]. We next performed an *in vitro* assay for avelumab-mediated ADCC to assess the functional significance of PD-L1 in chordoma cell lines (Figure 2). Avelumab increased NK-cell lysis 3.1-fold (*P* = 0.01) in JHC7 cells relative to isotype control. Similarly, avelumab increased NK-cell lysis relative to isotype control in UM-Chor1 (3-fold; *P* = 0.016), U-CH2 (1.7-fold; *P* = 0.029) and MUG-Chor1 cells (1.7-fold; *P* = 0.006). When the cells were treated with IFN-γ, avelumab markedly enhanced NK-cell lysis relative to isotype control in the following cell lines: JHC7 (7.56-fold; *P* = 0.001), UM-Chor1 (7.34-fold; *P <* 0.001), U-CH2 (2.6 fold; *P* = 0.008), MUG-Chor1 (8.38-fold; *P* = 0.0016). NK-cell lysis *via* ADCC occurs when CD16 (FcγRIII) on NK effector cells interacts with the Fc portion of antibodies recognizing target cells [28]. The addition of CD16 neutralizing antibody inhibited NK-cell lysis in all the cell lines, indicating that NK-cell lysis was mediated by ADCC (Figure 2). In sum, avelumab increases chordoma cells' sensitivity to NK-cell lysis *via* ADCC, and avelumab's efficacy is enhanced in chordoma cells that have IFN-γ-induced overexpression of PD-L1.

### Tumor antigen-specific CD8^+^ T cells increased avelumab-mediated sensitivity to ADCC

**Figure 3:**
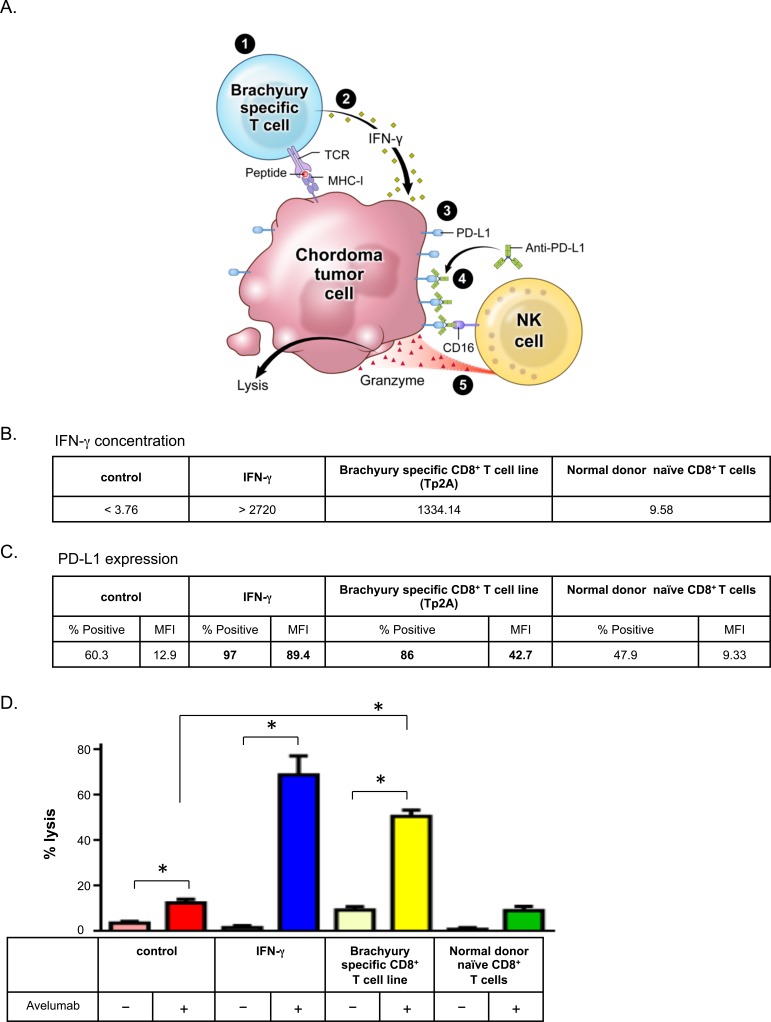
Brachyury-specific CD8^+^ T cells increased chordoma cells' sensitivity to avelumab-mediated ADCC **A.** Model of indirect enhancement of ADCC by tumor antigen-specific T cells: 1) brachyury-specific T-cell recognition of chordoma tumor; 2) induced IFN-γ secretion; 3) PD-L1 upregulation; 4) increased binding of anti-PD-L1 (avelumab); and 5) enhanced NK cell-mediated killing of tumor (ADCC). UM-Chor1 cells were co-cultured for 24 h with brachyury-specific CD8^+^ T cells or naïve T cells isolated from PBMCs of a normal donor. As a positive control, UM-Chor1 cells were treated or untreated with 50 ng/mL of IFN-γ for 24 h. **B.** Concentration of IFN-γ (pg/mL) in supernatant fluid following T cell/tumor cell co-incubation. **C.** Expression of PD-L1 in UM-Chor1 cells was analyzed by flow cytometry. Values in bold denote an increase of > 10% relative to vehicle control cells. **D.** ADCC assays were performed using UM-Chor1 cells, with purified normal donor NK cells as effector cells. Select groups of cells were incubated with avelumab. Statistical analyses were done by Student's *t* test. * = *P* < 0.05, error bars indicate mean ± SD for triplicate measurements. This experiment was repeated at least 2 times with similar results.

Having seen that IFN-γ treatment upregulated PD-L1 expression (Figure 1C) and increased sensitivity to ADCC (Figure 2), we sought to establish a model where IFN-γ would be produced by tumor antigen-specific CD8^+^ T cells. We hypothesized that IFN-γ released by tumor antigen-specific CD8^+^ T cells, either endogenous or vaccine-mediated, would upregulate PD-L1 expression on tumor cells, increasing their sensitivity to avelumab-mediated ADCC (Figure 3A). To test this hypothesis, we first co-cultured tumor cells and tumor antigen-specific CD8^+^ T cells. Chordoma cells have been shown to express the transcription factor brachyury [29, 30], especially the UM-Chor1 cell line [19], which we confirmed (Supplemental Figure 2). To examine the response of brachyury-specific CD8^+^ T cells to chordoma cells, we co-cultured HLA-A2^+^ UM-Chor1 cells for 24 h with brachyury-specific HLA-matched CD8^+^ T cells or naïve CD8^+^ T cells isolated from HLA-matched peripheral blood mononuclear cells (PBMCs) from a normal donor as control CD8^+^ T cells. Brachyury-specific CD8^+^ T cells increased IFN-γ production 140-fold (1334.14 pg/mL) over naïve CD8^+^ T cells (Figure 3B). Moreover, interaction with brachyury-specific CD8^+^ T cells increased PD-L1 expression in UM-Chor1 cells 3.31-fold; naïve CD8^+^ T cells did not show increased PD-L1 expression (Figure 3C). Finally, we performed ADCC assays using co-cultured UM-Chor1 cells to assess the effect of CD8^+^ T cells on their sensitivity to avelumab-mediated ADCC (Figure 3D). IFN-γ-treated cells increased avelumab-mediated ADCC 5.5-foldover untreated cells (*P* < 0.0001). Similarly, UM-Chor1 cells co-cultured with brachyury-specific CD8^+^ T cells increased avelumab-mediated ADCC 4.03-fold over untreated cells (*P* < 0.0001). Taken together, these results suggest that brachyury-specific CD8^+^ T cells increase PD-L1 expression on chordoma cells *via* IFN-γ production, increasing chordoma cells' sensitivity to avelumab-mediated ADCC.

### Phenotypic signature of a residential CSC population in chordoma cell lines

**Figure 4:**
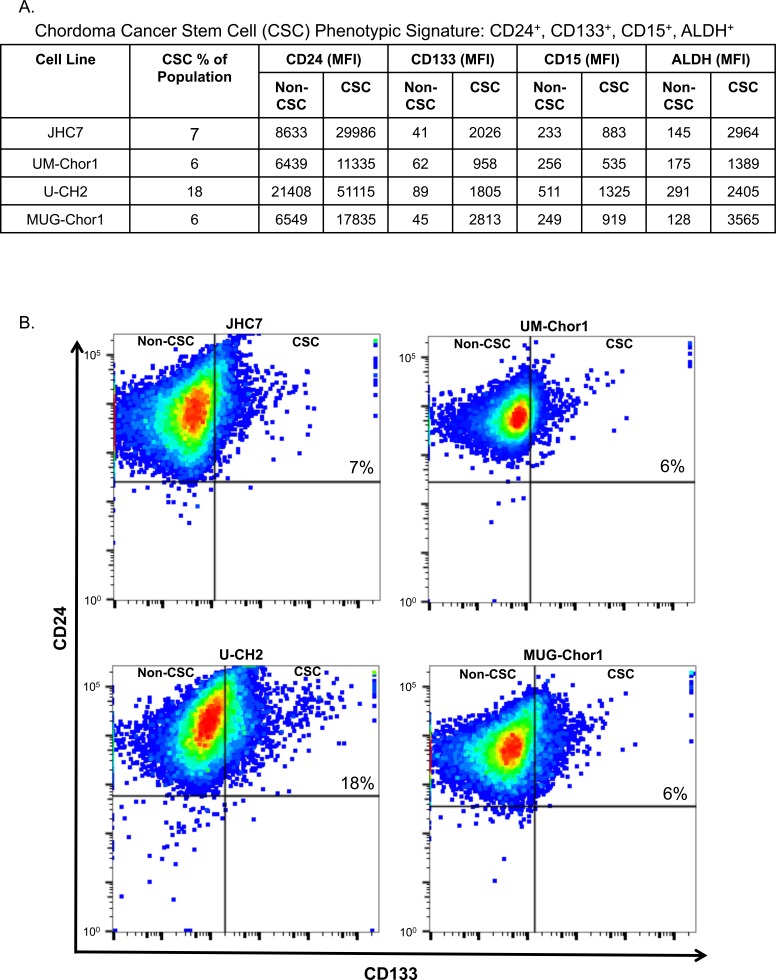
Phenotypic signature of a residential CSC population in chordoma cell lines Four chordoma cell lines were analyzed for expression of CD24, CD133, CD15, and ALDH by flow cytometric analyses. **A.** Respective MFIs for each CSC marker studied. This experiment was repeated 3 times with similar results. **B.** Representative expression of CD24 and CD133 for each of the 4 chordoma cell lines.

CSCs have been recognized in recent years as important players in the development of solid tumors. Cells with CSC characteristics are resistant to current treatment modalities including radiation and chemotherapy and are associated with poor treatment response rates and disease recurrence. Certain established tumor cell lines have been reported to harbor residential CSC populations. Previous studies have defined the CSC population in a single chordoma cell line, U-CH1, as expressing CD15 and CD133 [31]. We investigated the relative expression levels (mean fluorescence intensity; MFI) of CD24, CD133, CD15, and ALDH in the CD24^high^/CD133^high^ group in 4 chordoma cell lines (Figure 4A). Both CD15 and ALDH were markedly increased in the CD24^high^/CD133^high^ group, defined as the residential CSC population, compared to the non-CSC population (Figure 4A). As an example, the non-CSC population in JHC7 cells were CD24 (8633), CD133 (41), CD15 (233), and ALDH (145). These MFIs were markedly less than the MFIs observed in the CSC population. We observed similar MFIs for the non-CSC populations in the other chordoma cell lines. A residential CSC population was detectable in 4 of 4 cell lines, ranging from 6%-18% of the total population, as determined by CD24 and CD133 co-expression (Figure 4B). These data suggest that chordoma cells have a CSC subpopulation that can be identified by the stem cell markers CD24, CD133, CD15, and ALDH.

### Treating chordoma cells with IFN-γ increases NK-cell killing of both CSC and non-CSC populations *via* ADCC

**Figure 5:**
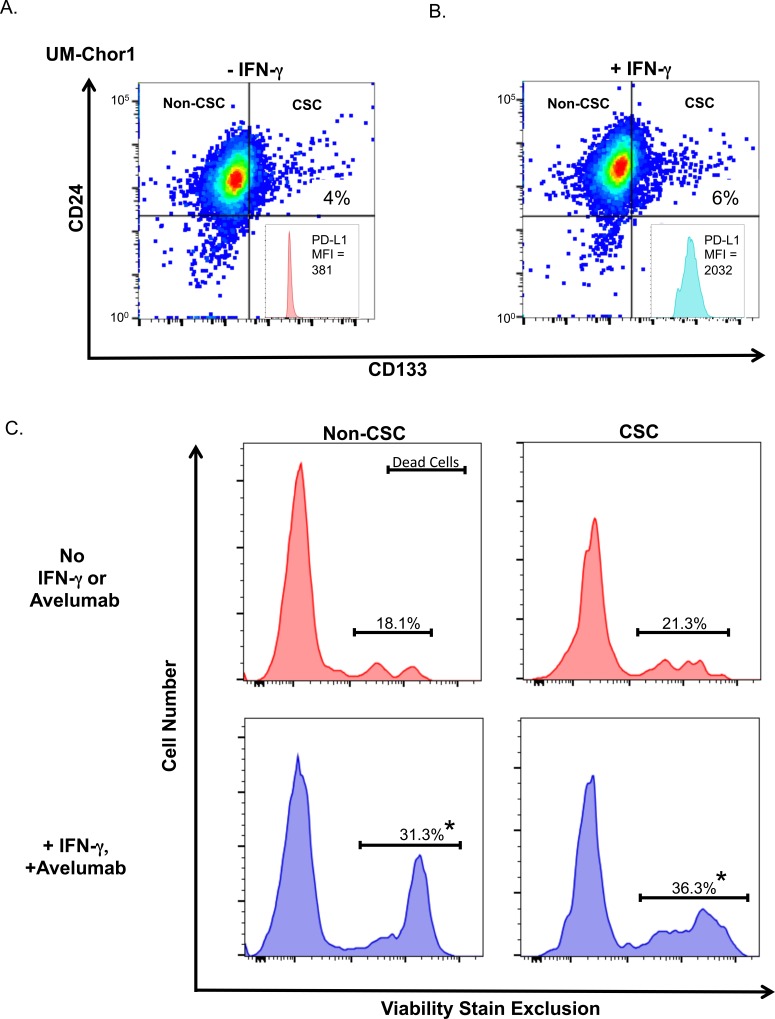
Treating chordoma cells with IFN-γ increases NK-cell killing of both non-CSC and CSC populations *via* ADCC Flow expression of CD24 and CD133 in a CSC subpopulation of UM-Chor1 cells treated **A.** without or **B.** with IFN-γ. Inset panels: PD-L1 expression on CSC populations. **C.** UM-Chor1 cells were (upper; red) untreated as baseline control or (lower; blue) treated with IFN-γ and underwent avelumab-mediated ADCC assay. Cells were stained with a live/dead viability marker to determine cell death in non-CSC and CSC populations. * = statistical significance relative to control (*P* < 0.05).

To determine whether avelumab-mediated ADCC could increase CSC subpopulation killing, we stained UM-Chor1 cells with the CSC markers CD24 and CD133 and treated them with or without IFN-γ. Treatment with IFN-γ did not change the frequency of the CSC subpopulation (Figure 5A, 5B). Flow cytometric staining analyses of these cells showed a 5-fold increase in PD-L1 expression following IFN-γ treatment (Figure 5B, inset). UM-Chor1 cells treated with IFN-γ were then subjected to an ADCC assay with avelumab, and the degree of cell death was determined by viability stain exclusion (Figure 5C). In this assay, we used untreated UM-Chor1 cells as a baseline for comparison to IFN-γ-treated UM-Chor1 cells that underwent ADCC. In the non-CSC group, ADCC-mediated cell death was 1.7-fold higher (Figure 5C; *P* < 0.001) than baseline cell death. Notably, ADCC-mediated cell death in the CSC group also had a significant increase (1.7-fold; *P* < 0.001) compared to cell death in the baseline CSC group. These data suggest that IFN-γ increases PD-L1 expression in the CSC subpopulation of chordoma cells, and that avelumab effectively increases ADCC of both the non-CSC and CSC subpopulations to the same degree.

## DISCUSSION

Immunotherapy has become a standard treatment for patients with certain cancers. The PD-1 inhibitors nivolumab and pembrolizumab are FDA-approved for melanoma and lung cancer. Currently, many clinical trials of agents that block the PD-1/PD-L1 pathway are ongoing in a range of cancers. The finding of a significant correlation between PD-L1 expression levels in tumor tissue and responsiveness to PD-1 pathway blockade [10, 32] has led to the investigation of PD-L1 expression in a variety of cancers, with the goal of developing further applications of PD-1 pathway blockade [33-36]. Feng *et al.* reported that PD-L1 is expressed in chordoma tissue samples, especially metastatic tumors [14]. It has also been reported that PD-L1 expression can be upregulated by IFN-γ in several cancers [12, 37]. Previous studies showed that IFN-γ could induce PD-L1 expression in the chordoma cell lines U-CH1, U-CH2, CH22, and JHC7 [14, 15]. Here, we confirmed previous reports that PD-L1 expression was upregulated by IFN-γ in U-CH2 and JHC7 (Figure 1) [14, 15]. We extended these observations to include the chordoma cell lines UM-Chor1 and MUG-Chor1, and confirmed that they also expressed PD-L1, which was upregulated by IFN-γ (Figures 1, 3, and 5).

The finding of PD-L1 expression in chordoma (Figure 1) suggested potential clinical benefit from PD-1/PD-L1 pathway blockade. Several anti-PD-L1 antibodies have been generated and are being evaluated in ongoing phase II/III clinical trials. Of these agents, avelumab appears to be the only one that induces ADCC in NK cells. Previously, our group reported that avelumab enhanced ADCC in various cancer cell lines that express PD-L1 [13]. However, the potential of anti-PD-L1 antibody therapy for ADCC in chordoma has not previously been shown. It should be noted that chordoma cells expressed a relatively high baseline expression of PD-L1 which could be further increased with IFN-γ (Figure 1). It has been reported that the overall cell-surface surface density of PD-L1 (as determined by, MFI) rather than the percentage of positive cells may be greater predictor or sensitivity to ADCC. Boyerinas et al., conducted flow cytometric analysis of a panel of 18 human tumor cell lines encompassing five different tumor types and showed that human carcinoma cell lines express a broad range of PD-L1 % positive cells and PD-L1 cell surface densities. Moreover, those cell lines with the highest cell surface expression were the most sensitive to ADCC mediated by avelumab [13].

Here, we show that avelumab significantly increased NK-cell lysis *via* ADCC in 4 of 4 chordoma cell lines (Figure 2). Moreover, avelumab's efficacy is further enhanced in chordoma cells where treatment with IFN-γ has induced overexpression of PD-L1 (Figure 2). It has also been reported that IFN-γ produced by activated CD8^+^ T cells can upregulate PD-L1 expression on tumor cells [38, 39]. A potential strategy for activating tumor-recognizing T cells is a vaccine encoding a tumor-specific antigen. Since chordoma expresses the transcription factor brachuyury [30], a rational strategy for treating chordoma may be a vaccine that encodes brachyury.

Historically, transcription factors have been considered undruggable targets [40] for standard cytotoxic agents, but other potential means of targeting brachyury have been proposed, including small inhibitory RNA, epigenetic modulation, and immune-based therapy [41, 42]. To date, the only attempts to target brachyury have been with 2 therapeutic cancer vaccines. Our group recently completed a phase I trial of GI-6301 (recombinant yeast-brachyury vaccine) that enrolled 11 patients with advanced chordoma [13]. An ongoing phase II clinical trial in chordoma patients is evaluating a brachyury vaccine in combination with radiation therapy. One patient had a confirmed radiographic partial response by RECIST, another had a mixed response, and the median progression-free survival in the chordoma group was 8.3 months. The vaccine induced brachyury-specific T-cell responses in the majority of all patients enrolled and in the subset of patients enrolled with chordoma. There were no significant vaccine-related toxicities [43]. Notably, the 2 patients with evidence of tumor shrinkage both had radiotherapy within 3.5 months of enrolling on study. Given the low likelihood of radiographic response with radiotherapy alone in advanced-stage disease, our hypothesis was that radiotherapy had an immunomodulatory effect on chordoma cells, making them more amenable to T cell-mediated killing.

To model a patient receiving a brachyury vaccine (Figure 3A), we co-incubated chordoma cells with brachyury-specific CD8^+^ T cells. The brachyury-specific CD8^+^ T cells recognized chordoma cells and increased PD-L1 expression on chordoma cells through the production of IFN-γ (Figure 3B and 3C). This increased PD-L1 expression significantly increased the chordoma cells' sensitivity to avelumab-mediated ADCC of NK cells (Figure 3D). In a previous study we observed that radiation did not modulate PD-L1 expression on tumor cell lines [44]. Similarly, chordoma cell lines (JHC7, UM-Chor1, U-CH2, and MUG-Chor1) exposed to 8 Gy radiation showed no increase in PD-L1 expression after 72 h (Supplemental Table 1). Radiation therapy has been reported to upregulate PD-L1 on tumors *in-vivo*, likely indirectly from the radiation induced inflammatory response. Future studies will focus on how to exploit PD-L1 modulation in response to radiation. These observations suggest a rationale for treating additional cohorts of the ongoing phase II trial (NCT02383498) with avelumab.

It has been shown that MHC-I expression is increased by IFN-γ and upregulated in cancer tissue [16, 17]. Our study showed that IFN-γ also increased expression of HLA-ABC in chordoma cells (Figure 1C). While some have suggested that increased MHC-I expression may induce resistance to NK-cell lysis, as NK cells discriminate between self and non-self by monitoring the expression of MHC-I molecules [45], increased expression of MHC-I has actually been shown to enhance sensitivity to cytotoxic T lymphocytes (CTLs) by upregulating antigen processing and presentation on tumor cells [17]. Our results indicate that chordoma cells treated with vaccine may have increased sensitivity not only to ADCC, but also to CTLs.

We assessed the molecular consequences of IFN-γ-induced gene expression by microarray analysis, focusing on UM-Chor1, which showed the greatest increase of IFN-γ-induced PD-L1 expression among the 4 chordoma cell lines (Figure 1C). The gene with the second highest upregulation, *CEBPD*, is a transcription factor that modulates many biological processes, including cell differentiation, motility, growth arrest, proliferation, and cell death. Though it was reported that *CEBPD* was induced by IFN-γ in certain cancers [24, 46], *CEBPD* functions both as a tumor suppressor and a tumor promoter [47]. Here we showed the predicted pathway of PD-L1 expression induced by IFN-γ, deduced from the results of microarray analysis. *CEBPD* is induced by IFN-γ, which leads to inhibition of *MYC* and activation of *TLR9*, *IL10*, and *TNF*, and culminates in upregulation of PD-L1 (*CD274*) expression (Supplemental Figure 1C). This suggests that *CEBPD* is potentially involved in the pathway of IFN-γ-induced PD-L1 expression in chordoma cells. This would block the innate immune response and result in tumor progression. Further investigation is needed to confirm the function of *CEBPD* in chordoma cells.

Recently, CSCs have been recognized as critical to the development of solid tumors. CSCs have been reported to be largely resistant to treatments such as radiation and chemotherapy [48]. There have been concerted efforts to determine the markers that define CSCs in several cancers. It is noteworthy that the expression of CSC surface markers is tissue type-specific. For example, the CSC surface markers CD44^+^ and CD24^−^ were defined in breast cancer, CD20^+^ and ABCB5^+^ in melanoma, and EpCAM^+^, CD44^+^, and CD166^+^ in colon cancer [49]. In an effort to define the phenotypic signature of chordoma CSCs, we observed that CD15 and ALDH were also highly expressed in CD24^high^/CD133^high^ (Figure 4A). ALDH has been widely used as a CSC marker in various cancer types [50]. Thus, we found that CD24^high^/CD133^high^ cells could be defined as a CSC subpopulation in chordoma cells. Moreover, CD24^high^/CD133^high^ cells showed high expression of CD15. These data confirm and extend previous findings that CD15 and CD133 were candidates for CSC markers in chordoma, using a U-CH1 cell line [31]. Our data suggest that, as the stem markers, CD24, CD133, CD15, and ALDH could identify a residential CSC subpopulation in chordoma. However, the potential of immunotherapy for CSCs remains controversial [51]. Previous studies showed that breast CSCs had resistance to NK killing due to reduced expression of the ligands for NKG2D, the stimulatory NK cell receptor [52]. On the other hand, Tallerico *et al.* reported that colorectal CSCs express higher levels of ligand for the natural cytotoxicity receptors that mediate NK-cell killing [53]. Thus, we investigated the possibility of treating chordoma CSCs with immunotherapy. IFN-γ treatment upregulated PD-L1 expression in the CSC population of chordoma (Figure 5A, 5B), suggesting PD-L1 blockade as a potential treatment for chordoma CSCs. Furthermore, the chordoma CSCs were killed by avelumab-mediated ADCC to the same degree as the non-CSCs (Figure 5C).

Our study is the first to show that PD-L1 expression induced by IFN-γ increases chordoma cells' sensitivity to NK-cell lysis *via* avelumab-mediated ADCC. Moreover, in a model of a patient receiving a tumor antigen-specific vaccine, brachyury-specific CD8^+^ T cells increased PD-L1 expression on chordoma cells through the production of IFN-γ, increasing the sensitivity of chordoma cells to avelumab-mediated ADCC. We also identified the residential CSC population in chordoma cells and showed that they were killed by avelumab-mediated ADCC. Our findings indicate the potential of avelumab to enable endogenous NK cells to kill chordoma cells *via* ADCC, as well as the potential of combination therapy, such as a T-cell vaccine and avelumab, to enhance NK-cell killing of chordoma cells *via* ADCC. Our findings suggest that while chordoma is resistant to conventional therapies such as radiotherapy and chemotherapy, immune-mediated therapy may have clinical benefit for patients with chordoma.

## MATERIALS AND METHODS

### Cell culture and reagents

Chordoma cell lines JHC7 and UM-Chor1 were obtained from the Chordoma Foundation (Durham, NC). The chordoma cell lines U-CH2 (ATCC^®^ CRL-3218^TM^) and MUG-Chor1 (ATCC^®^ CRL-3219^TM^) were obtained from American Type Culture Collection (Manassas, VA). All cells were passaged for fewer than 6 months. JHC7 cells were maintained in DMEM/F12 medium supplemented with 10% fetal bovine serum and 1% penicillin/streptomycin. UM-Chor1 cells were maintained in Iscove's modification of DMEM and RPMI1640 medium (4:1), supplemented with 10% fetal bovine serum, 1% penicillin/streptomycin, and nonessential amino acids. U-CH2 and MUG-Chor1 cells were maintained in Iscove's modification of DMEM and RPMI1640 medium (4:1), supplemented with 10% fetal bovine serum and 1% penicillin/streptomycin. In addition, MUG-Chor1 cells required 10 μg/mL human insulin. PBMCs from healthy donors were obtained from the NIH Clinical Center Blood Bank (NCT00001846).

The anti-PD-L1 mAb avelumab and matching IgG1 isotype control were obtained from EMD Serono as part of a Cooperative Research and Development Agreement with the Laboratory of Tumor Immunology and Biology, National Cancer Institute.

### Flow cytometry

To assess the effect of IFN-γ on the cell-surface phenotype of chordoma cells, cells were untreated or treated with 50 ng/mL of IFN-γ (R&D Systems, Minneapolis, MN) for 24 h. Cells were then harvested and stained with the following antibodies: HLA-ABC-FITC (BD Biosciences, San Jose, CA), PD-L1-APC (clone 29E.2A3; BioLegend, San Diego, CA), CD133-PE (Miltenyi Biotec, San Diego, CA), CD24-PerCP-Cy 5.5 (BD Biosciences), CD15-V450 (BD Biosciences), and ALDHA1-FITC (USBiological, Salem, MA). Cell viability was examined using far red fluorescent reactive dye (Thermo Fisher, Waltham, MA). Cells were incubated with the antibodies for 30 min at 4°C, acquired on a FACSCalibur flow cytometer or FACSVerse (Becton Dickinson, Franklin Lakes, NJ), and analyzed using FlowJo software (TreeStar, Inc., Ashland, OR). Isotype control staining was < 5% for all samples analyzed.

### Microarray analysis and statistical analysis

UM-Chor1 cells were left untreated or treated with 50 ng/mL of IFN-γ for 24 h. Cells were then harvested and total RNA was isolated using the RNAeasy Plus minikit (Qiagen, Valencia, CA). 100 ng of RNA was reverse transcribed and amplified using a WT expression kit (Ambion, Austin, TX). Sense strand cDNA was fragmented and labeled using a WT terminal labeling kit (Affymetrix, Santa Clara, CA). Three replicates of each group were hybridized to the Human Gene ST 2.0 GeneChip (Affymetrix) and scanned on the GeneChip scanner 3000 (Affymetrix). Data were collected using Affymetrix AGCC software.

Microarray data are available in the GEO under accession number GSE77732. Statistical and clustering analysis for the microarray experiment was performed with Partek Genomics Suite software (St. Louis, MO) using an RMA normalization algorithm. Differentially expressed genes were identified by ANOVA. Genes that were up- or downregulated > 1.5-fold with a *P* < 0.05 were considered significant. Significant genes were analyzed for enrichment of pathways using Ingenuity Pathway Analysis software (Qiagen, Redwood City, CA).

### Antibody-dependent cellular cytotoxicity assay

The ADCC assay was performed as previously reported [13] with indicated modifications. Cells were left untreated or treated with 50 ng/mL of IFN-γ for 24 h. Cells were then harvested and labeled with ^111^In. Cells were plated as targets at 2,000 cells/well in 96-well round-bottom culture plates and incubated with 2 μg/mL of avelumab or control isotype antibody at room temperature for 30 min. NK cells were added at 100,000 cells/well at an effector-to-target (E:T) ratio of 50:1. After 4 h, supernatants were harvested and analyzed for the presence of ^111^In using a WIZARD2 Automatic Gamma Counter (PerkinElmer, Waltham, MA). Spontaneous release was determined by incubating target cells without effector cells, and complete lysis was determined by incubation with 0.05% Triton X-100. Experiments were carried out in triplicate. Specific ADCC lysis was determined using the following equation: Percent lysis = [(experimental cpm - spontaneous cpm) / (complete cpm - spontaneous cpm)] x 100.

To verify that CD16 (FcγRIII) on NK cells engage avelumab-mediated ADCC, CD16 mAb was used to block CD16. NK cells were incubated with 2 μg/mL of CD16 mAb (clone B73.1; eBioscience, San Diego, CA) for 2 h before being added to target cells.

To examine the relationship between a CSC subpopulation and ADCC activity, UM-Chor1 cells were left untreated or treated with 50 ng/mL of IFN-γ for 24 h. Cells were then plated as targets at 50,000 cells/well in 6-well round-bottom culture plates and incubated with 2 μg/mL of avelumab at room temperature for 30 min. NK cells were added at 2500,000 cells/well at an E:T ratio of 50:1. After 4 h, tumor cells were harvested and stained with antibodies for flow cytometry.

### Co-culture of chordoma cells and T cells

The HLA-A2-restricted brachyury-specific CD8^+^ CTL line (Tp2A) recognizes the brachyury epitope WLLPGTSTL (T-p2) [54]. Normal donor CD8^+^ T cells were isolated from normal donor HLA-A2^+^ PBMCs using the Human CD8^+^ T Cell Isolation (negative selection) Kit 130-096-495 (Miltenyi Biotec) per the manufacturer's protocol.

UM-Chor1 cells were co-cultured in 12-well plates with Tp2A or normal donor CD8^+^ T cells at a tumor cell/CD8^+^ T cell ratio of 2:1. As a positive or negative control, UM-Chor1 cells were left untreated or treated with 50 ng/mL of IFN-γ for 24 h. After a 24-h co-culture, the supernatant fluid was harvested and the concentration of IFN-γ was measured using a multiplex cytokine/chemokine kit (Meso Scale Discovery, Gaithersburg, MD). Tumor cells were harvested and used as a target for the ADCC assay, as described above, and stained with PD-L1 antibody for flow cytometry.

### Western blot analysis

The Western blot was performed as previously described [55] with indicated modifications. Protein lysate was extracted from UM-Chor1 cells. The primary antibodies used were monoclonal rabbit antibody (mAb 54-1, 1 μg/mL) against human brachyury [29] and GAPDH (Cell Signaling Technology, Danvers, MA).

### Statistical analysis

Significant differences in the distribution of data acquired by flow cytometry analysis were determined by the Kolmogorov-Smirnov test using FlowJo software (TreeStar, Inc.). Significant differences in the distribution of data acquired by ADCC assays were determined by paired Student's *t* test with a 2-tailed distribution and reported as *P* values, using Prism 6.0f software (GraphPad Software Inc., La Jolla, CA).

## SUPPLEMENTARY MATERIAL FIGURES AND TABLE


