# Supplementary material for: Enhanced killing of chordoma cells by antibody-dependent cell-mediated cytotoxicity employing the novel anti-PD-L1 antibody avelumab
Source: Oncotarget. 2016 May 9;7(23):33498–511. doi: 10.18632/oncotarget.9256 (PMC5085098; doi:10.18632/oncotarget.9256)
Supplement: Supplementary file 1 [file oncotarget-07-33498-s001.pdf]

Enhanced killing of chordoma cells by antibody-dependent cell-mediated cytotoxicity employing the novel anti-PD-L1 antibody avelumab

Supplementary Material

A.

Upregulated genes

Symbol	Name (additional names)	Fold (over control)	Function
TP53INP2	tumor protein p53 inducible nuclear protein 2 (DOR)	2.339	Bi-functional protein that regulates transcription and enhances starvation-induced autophagy.
CEBPD	CCAAT/enhancer binding protein (C/EBP), delta	1.986	Transcription factor that upregulates pro-inflammatory genes and is upregulated by IFN-γ.
UQCR11	ubiquinol-cytochrome c reductase, complex III subunit XI	1.725	Component of the ubiquinol-cytochrome c reductase complex - part of the mitochondrial respiratory chain.
SLC1a4	solute carrier family 1 (glutamate/neutral amino acid transporter), member 4 (SATT) or (ASCT1)	1.651	lanine, serine, cysteine transporter.
PSMA7	proteasome subunit alpha 7	1.578	Alpha-type subunit of the 20S proteasome core complex and participates in degrading proteins through ubiquitin-proteasome pathway (UPP) .
ADAP1	ArfGAP with dual PH domains 1, (GCS1L; CENTA1; p42IP4)	1.568	Functions as a scaffolding protein in several signal transduction pathways, including actin and microtubule cytoskeletal crosstalk.

B.

Downregulated genes

Symbol	Name	Fold (over control)	Function
CLDN2	claudin 2	-1.844	One of the best studied pore-forming claudins. Reduced expression correlates with a tigher cell to cell junctions.
PHACTR4 (STOP)	phosphatase and actin regulator 4	-1.694	Restrains normal cell proliferation and transformation. Depletion of Phactr4 leads to increased proliferation.
SEPP1	selenoprotein P, plasma, 1	-1.641	A heparin-binding protein that appears to be associated with endothelial cells, and has been implicated to function as an antioxidant in the extracellular space.
TYR	tyrosinase	-1.607	Enzyme involved in the conversion of tyrosine to melanin. Potentially involved in MHC Presentaiton
TMEM63A	Transmembrane protein 63A	-1.588	Unknown
CNP	2',3'-cyclic nucleotide 3' phosphodiesterase	-1.583	Regulates multiple cellular functions and suppress protein production by association with polyadenylation of mRNA
PGM2	phosphoglucomutase 2	-1.558	Catalyzes the conversion of the nucleoside breakdown products.

C.

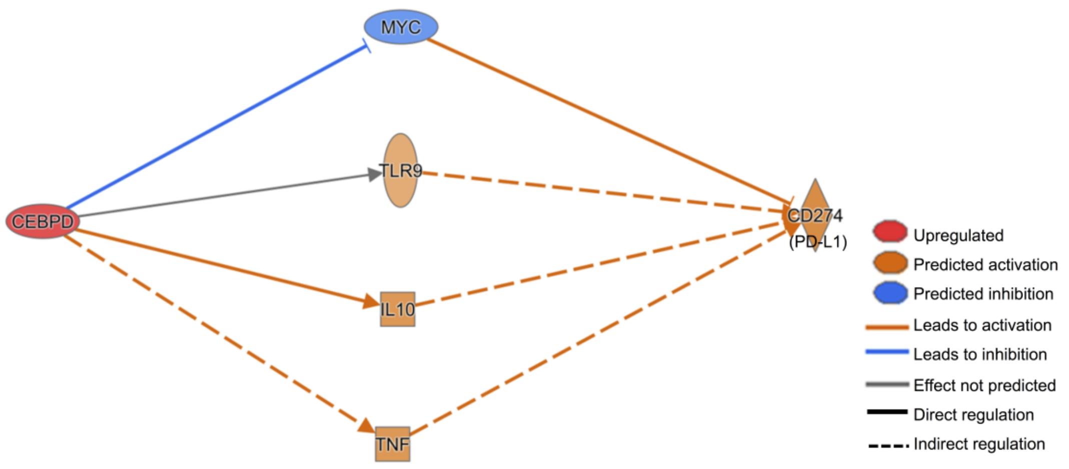

Supplemental Figure 1: Expression profiles of IFN-γ-treated genes in UM-Chor1 cells. Gene expression profiles of IFN-γ-treated UM-Chor1 cells and untreated control cells were assessed by microarray analysis. **A.** Genes significantly up- or **B.** downregulated by IFN-γ (> 1.5-fold over control cells; $P < 0.05$). Gene symbol, name, fold over control cells, and annotated function are shown. **C.** IPA prediction analysis performed using the significantly affected unfolded protein response-related genes. Schematic demonstrates the potential relationship between the transcription factor CEBPD and PD-L1 (CD274).

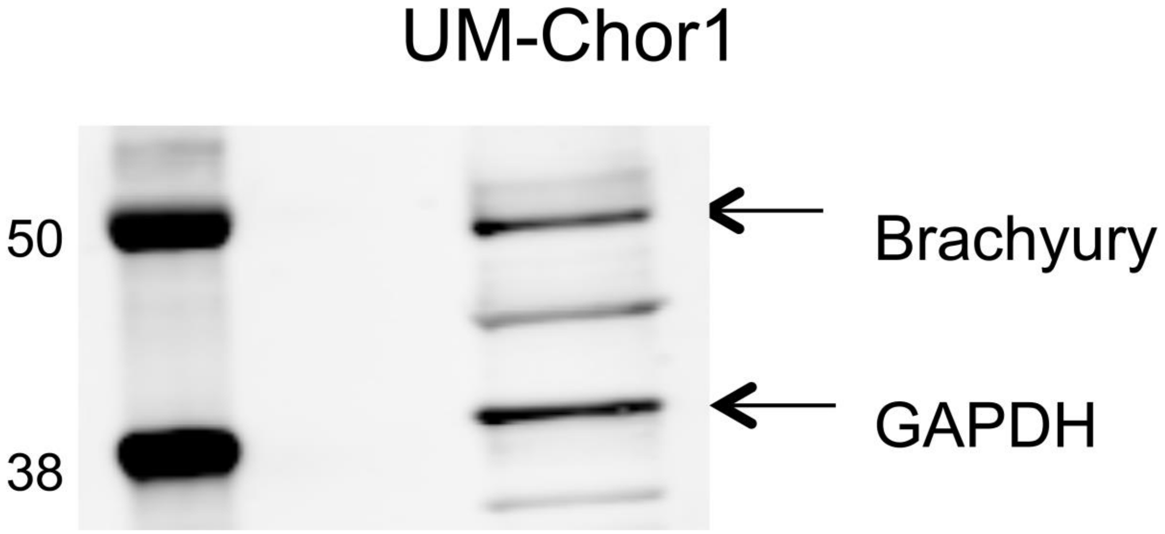

Supplemental Figure 2: Expression of brachyury protein in UM-Chor1 chordoma cells by Western blot analysis.

Supplemental Table 1: Radiation exposure does not upregulate PD-L1 in chordoma cells.

Surface expression of PD-L1

	**Non-Radiation**		**8Gy-Radiation**	
	**%positive**	**MFI**	**%positive**	**MFI**
JHC7	91	176	78	171
UM-Chor1	60	24	28	19
U-CH2	95	186	88	161
MUG-Chor1	10	14	10	23

Suspensions of chordoma cells were irradiated with 8 Gy by exposure to a Cs-137 source (Gammacell-1000; AECL/Nordion; Kanata, Ontario, Canada). After 72 hours, cells were harvested and analyzed by flow cytometry. PD-L1 percent positivity and mean fluorescence intensity (MFI) of chordoma cells with/without radiation are presented.
